# Crystal structure of the Fe-member of usovite

**DOI:** 10.1107/S2056989015009251

**Published:** 2015-05-20

**Authors:** Matthias Weil

**Affiliations:** a Institute for Chemical Technologies and Analytics, Division of Structural Chemistry, Vienna University of Technology, Getreidemarkt 9/164-SC, A-1060 Vienna, Austria

**Keywords:** crystal structure, usovite, Fe-containing usovite, disorder, fluorides

## Abstract

Crystals of the title compound, with the idealized composition Ba_2_CaFeAl_2_F_14_, dibarium calcium iron(II) dialuminium tetra­deca­fluoride, were obtained serendipitously by reacting a mixture of the binary fluorides BaF_2_, CaF_2_ and AlF_3_ in a leaky steel reactor. The compound crystallizes in the usovite structure type (Ba_2_CaMgAl_2_F_14_), with Fe^2+^ cations replacing the Mg^2+^ cations. The principal building units are distorted [CaF_8_] square-anti­prisms (point group symmetry 2), [FeF_6_] octa­hedra (point group symmetry -1) and [AlF_6_] octa­hedra that are condensed into undulating ^2^_∞_[CaFeAl_2_F_14_]^4−^ layers parallel (100). The Ba^2+^ cations separate the layers and exhibit a coordination number of 12. Two crystal structure models with a different treatment of the disordered Fe site [mixed Fe/Ca occupation, model (I), *versus* underoccupation of Fe, model (II)], are discussed, leading to different refined formulae Ba_2_Ca_1.310 (15)_Fe_0.690 (15)_Al_2_F_14_ [model (I)] and Ba_2_CaFe_0.90 (1)_Al_2_F_14_ [model (II)].

## Abstract 2

The compound with the idealized composition Ba_2_CaFeAl_2_F_14_ crystallizes in the usovite structure type. Two models with different treatment of the disordered Fe site are presented.

## Chemical context   

Fluorido­aluminates with alkaline earth cations exhibit a rich crystal chemistry (Babel & Tressaud, 1985 ▸; Weil *et al.*, 2001 ▸). They are suitable host materials for optical applications as has been shown by luminescence exitation studies of SrAlF_5_ or CaAlF_5_ doped with Pr^3+^ and Mn^2+^ (van der Kolk *et al.*, 2004 ▸). In order to prepare large single crystals of a related fluorido­aluminate with composition BaCaAlF_7_, a different preparation route was chosen in comparison with the reported crystal-growth procedure. Instead of using a ZnCl_2_ melt (Werner & Weil, 2003 ▸), a carbon tool steel container shielded with a molybdenum foil was used for solid state reactions between a mixture of the binary fluorides (Weil & Kubel, 2002 ▸). However, during one of these experiments it turned out that the container was not completely lined by the molybdenum foil which consequently led to a reaction with the container wall and an incorporation of iron into parts of the reaction products. Crystal structure analysis of selected crystals from this reaction batch revealed an Fe-containing phase that crystallizes isotypically with the mineral usovite, Ba_2_CaMgAl_2_F_14_ (Litvin *et al.*, 1980 ▸).

Compounds with the usovite-type structure are represented by the general formula Ba_2_(*M* ^II^1)(*M* ^II^2)(*M* ^III^3)_2_F_14_ (*M* ^II^1 = Ca, Cd, Mn; *M* ^II^2 = Mg, Co, Mn, Cu, Cd, Fe; *M* ^III^3 = Al, V, Fe, Cr, Ga, Mn) and crystallize with four formula units in the space group *C*2/*c*. Most of the usovite-type representatives known so far were prepared and structurally determined by Babel, Tressaud and co-workers over the last three decades (Holler *et al.*, 1984 ▸, 1985 ▸; Kaiser *et al.*, 2002 ▸; Le Lirzin *et al.*, 1990 ▸, 1991 ▸, 2008 ▸; Qiang *et al.*, 1988 ▸).

## Structural commentary   

**Figure 1:**
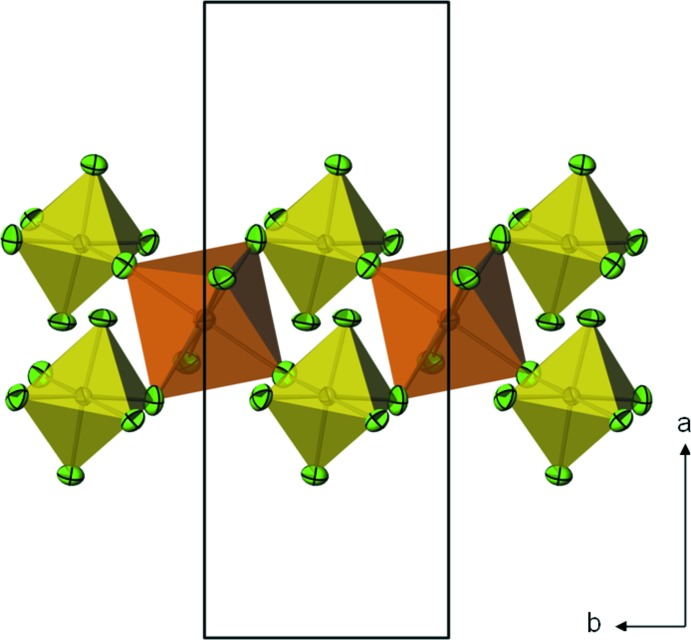
[AlF_6_] octa­hedra (yellow, with F atoms green) and [FeF_6_] octa­hedra (orange) are linked into crosslinked double chains parallel to [010]. Displacement ellipsoids are drawn at the 74% probability level.

**Figure 2:**
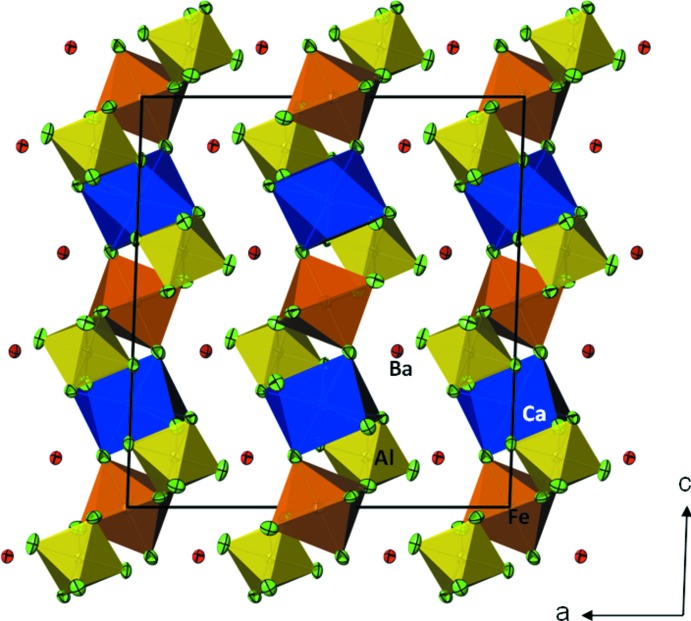
The crystal structure of the usovite-type title compound, emphasizing the formation of the layered ^2^_∞_[CaFeAl_2_F_14_]^4−^ framework parallel to (100), separated by Ba^2+^ cations. Displacement ellipsoids are drawn at the 74% probability level. The colour code is as in Fig. 1 ▸, with [CaF_8_] polyhedra in blue and Ba atoms in red.

The principal building units of the usovite crystal structure are distorted [BaF_12_] polyhedra, [(*M* ^II^1)F_8_] sqare-anti­prisms (point group symmetry 2) and [(*M* ^II^2)F_6_] octa­hedra (point group symmetry 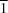), as well as rather regular [(*M* ^III^3)F_6_] octa­hedra. The [(*M* ^II^2)F_6_] and [(*M* ^III^3)F_6_] octa­hedra are connected by corner-sharing into infinite crosslinked double chains ^1^_∞_[(*M* ^II^2)F_2_F_4/2_(*M* ^III^3)_2_F_8_F_4/2_] extending parallel to [010] (Fig. 1 ▸). Neighbouring chains are linked by the [(*M* ^II^1)F_8_] square-anti­prisms into undulating (100) layers with composition ^2^_∞_[(*M* ^II^1)(*M* ^II^2)(*M* ^III^3)_2_F_14_)]^4−^, with the Ba^2+^ cations separating the individual layers (Fig. 2 ▸).

The unit-cell volume of the title compound [1067.9 (2) Å^3^] is slightly larger than that of usovite Ba_2_CaMgAl_2_F_14_ (1027.9 Å; Litvin *et al.*, 1980 ▸) due to the replacement of the Mg^2+^ cations (ionic radius = 0.72 Å; Shannon, 1976 ▸) by the larger Fe^2+^ cations (ionic radius = 0.78 Å; Shannon, 1976 ▸) at the *M* ^II^2 site. This is also reflected by the bond lengths within the individual coordination polyhedra (Table 1 ▸). Wheras the Ba—F, Ca—F and Al—F distances remain nearly unaltered between the two structures, the Mg—F and Fe—F distances show remarkable differences. The Mg—F distances in the usovite structure range from 1.939 to 2.041 Å, the corresponding Fe—F distances in the title structure from 2.015 (2) to 2.216 (2) Å, with a mean distance of 2.123 Å. The latter is in reasonable agreement with the mean Fe^II^—F distance of 2.106 Å in the isotypic crystal structure of Ba_2_CaFeV_2_F_14_ (Kaiser *et al.*, 2002 ▸). However, the mean bond lengths in both the title structure and Ba_2_CaFeV_2_F_14_ are considerably longer than that of 2.074 Å in the structure of the binary compound FeF_2_ (Jauch *et al.*, 1993 ▸).

A similar increase of the *M*—F bond lengths of the [(*M* ^II^2)F_6_] octa­hedra was also observed for a series of other usovite-type structures and was associated with an occupational disorder of the *M* ^II^2 site. For these models, either a mutual substitution of Ca^2+^ (on the *M* ^II^1 site) with corresponding divalent transition metal ions on the *M* ^II^2 site, or partial replacement of the divalent transition metal ions by Ca^2+^ at the *M* ^II^2 site was considered, resulting in stoichiometric compounds and Ca-richer compounds, respectively (Kaiser *et al.*, 2002 ▸). In the case of the title compound, a model with mutual substitution of Ca^2+^ and Fe^2+^ on the *M* ^II^1 and *M* ^II^2 sites could be ruled out during refinement. However, a model with an incorporation of Ca^2+^ on the Fe^2+^ site resulted in a ratio of Ca:Fe = 0.155 (7):0.345 (7) for this site [model (I); overall refined formula for the compound: Ba_2_Ca_1.310 (15)_Fe_0.690 (15)_Al_2_F_14_] and converged with the same reliability factors and remaining electron densities as the model without an incorporation of Ca^2+^ and underoccupation of the Fe^2+^ site only [model (II); Table 3]. The refined formula for this model is Ba_2_Ca_2_Fe_0.90 (1)_Al_2_F_14_. Bond lengths and angles of the two models are the same within the corresponding standard uncertainties (Table 1 ▸).

Kaiser *et al.* (2002 ▸) have discussed in detail the pros and cons of the incorporation of Ca^2+^ (ionic radius = 1.0 Å; Shannon, 1976 ▸) at the *M* ^II^2 site for various usovite-type structures. Strong arguments supporting an *M* ^II^2 site with mixed Fe/Ca occupation are the resulting bond-valence sums (Brown, 2002 ▸) that deviate significantly from the expected values of 2 if only Fe^2+^ ions are considered to be present at the *M* ^II^2 site (Table 2 ▸). Contrariwise, the bond-valence sums are in excellent agreement with the expected value if a mixed Fe/Ca occupancy is taken into account. The corresponding numbers are listed in Table 2 ▸ and were calculated with the weighted average occupancy ratio of Fe:Ca = 0.77:0.23 that was estimated by the program *VaList* (Wills, 2010 ▸). This ratio is in good agreement with the occupancy ratio from the refinement [model (I): Fe:Ca = 0.69:0.31]. The resulting global instability index (Brown, 2002 ▸) of 0.04 valence units for model (I) suggests a very tightly bonded structure with little strain. Any strain inherent in the usovite structure is obviously relieved by the substitution of Ca^2+^ on the *M* ^II^2 site.

On the other hand, an *M* ^II^2 site without an incorporation of Ca^2+^ would result in an underoccupation of Fe^2+^ [model (II)] and consequently requires the presence of an element in a higher oxidation state (here most probably Fe^3+^) to compensate the negative charge of −2 of the [Ba_2_CaAl_2_F_14_] framework. Although in this case rather a decrease of *M* ^II^2—F bond lengths should be expected (contrary to the findings of the current study), it cannot competely ruled out that Fe^3+^ ions are present at this site. As a matter of fact, based on diffraction data alone, there is a clear tendency towards model (I) but no definite answer whether Fe is partly replaced by Ca on the *M* ^II^2 site [model (I)] or is statistically occupied by Fe^2+^ and small amounts of Fe^3+^ [model (II)]. Complementary analytical techniques like Mössbauer spectroscopy will be needed in future to shed some light on this problem.

## Synthesis and crystallization   

The binary fluorides AlF_3_ (Merck, Patinal), CaF_2_ (Merck, Supra­pur) and BaF_2_ (Riedel de Haen, pure) were mixed in the stoichiometric ratio 1:1:1 and thoroughly ground in a ball mill, pressed into tablets and placed in a carbon tool steel container shielded with a molybdenum foil. NH_4_F·HF (100 mg, Fluka, p·A.) were added to the mixture to increase the HF pressure, to expel the remaining oxygen and to adjust a slightly reducing atmosphere during the reaction. The reactor was then closed and heated to 1173 K in the course of 20 h, kept at that temperature for 24 h, and then cooled slowly to 973 K at a rate of 10 K h^−1^, kept at this temperature for 24 h and finally cooled to room temperature overnight. After opening the reactor it became evident that parts of the molybdenum foil were torn apart accompanied by a severe attack of the inner container wall. Single crystals of the title compound were separated from the obtained colourless to light-green bulk material. X-ray powder diffraction of the bulk revealed the formation of α-BaCaAlF_7_ as the main phase and the title compound as a minority phase. Some additional reflections were also present that could not be assigned to any known phases.

## Refinement   

Crystal data, data collection and structure refinement details are summarized in Table 3 ▸. Coordinates of usovite (Litvin *et al.*, 1980 ▸) were used as starting parameters for refinement. The model converged rather smoothly with *R*1 = 0.034 and *wR*2 = 0.089. However, negative residual electron density at the Fe atom pointed to an underoccupation and/or a statistical disorder of the *M* ^II^2 site with a lighter element present. In fact, free refinement of the site occupation factor for this site resulted in only 90% occupancy and significant better reliability factors (see Table 3 ▸). The same procedure applied for all other atoms resulted in full occupancy within the twofold standard uncertainty. For the final models, full occupancy was therefore considered for all atoms except Fe. Model (I) accounts for an incorporation of Ca^2+^ at the Fe site under consideration of full occupancy; in model (II), the site occupation factor of the Fe site was refined freely without contribution of Ca^2+^ at this site. The remaining electron densities (Table 3 ▸) are virtually the same for both models. They are associated with truncation effects close to the heavy Ba sites, with the maximum electron density 0.68 Å and the minimum electron density 0.96 Å away from the Ba atom.

## Supplementary Material

Crystal structure: contains datablock(s) modelI, modelII, global. DOI: 10.1107/S2056989015009251/br2250sup1.cif

Structure factors: contains datablock(s) modelI. DOI: 10.1107/S2056989015009251/br2250modelIsup2.hkl

Structure factors: contains datablock(s) modelII. DOI: 10.1107/S2056989015009251/br2250modelIIsup3.hkl

CCDC references: 1401194, 1401194, 1401195

Additional supporting information:  crystallographic information; 3D view; checkCIF report

## Figures and Tables

**Table 1:** Selected bond lengths (Å) for model (I) For model (II), bond lengths are the same within their standard uncertainties.

Ba—F7	2.696 (2)	Ca2—F4	2.376 (2)
Ba—F4^i^	2.730 (2)	Ca2—F4^ix^	2.376 (2)
Ba—F1^i^	2.755 (2)	Ca2—F5^x^	2.544 (2)
Ba—F2^ii^	2.765 (2)	Ca2—F5^iv^	2.544 (2)
Ba—F5^iii^	2.766 (2)	Fe1—F7^x^	2.015 (2)
Ba—F5^iv^	2.827 (2)	Fe1—F7^i^	2.015 (2)
Ba—F3^iv^	2.889 (2)	Fe1—F2^xi^	2.131 (2)
Ba—F1^v^	2.889 (2)	Fe1—F2^viii^	2.131 (2)
Ba—F3	2.974 (2)	Fe1—F3^x^	2.216 (2)
Ba—F6^iii^	3.101 (2)	Fe1—F3^i^	2.216 (2)
Ba—F6^iv^	3.158 (2)	Al—F4^xii^	1.780 (2)
Ba—F1^vi^	3.233 (3)	Al—F1	1.780 (3)
Ca2—F7^vii^	2.235 (2)	Al—F6^iv^	1.790 (2)
Ca2—F7^i^	2.235 (2)	Al—F2^iv^	1.799 (2)
Ca2—F6^iii^	2.369 (2)	Al—F5^iii^	1.843 (2)
Ca2—F6^viii^	2.369 (2)	Al—F3^iii^	1.846 (2)

Symmetry codes: (i) 
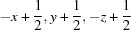
; (ii) 
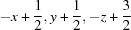
; (iii) 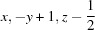; (iv) 
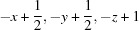
; (v) 
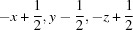
; (vi) 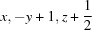; (vii) 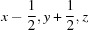; (viii) 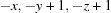; (ix) 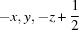; (x) 
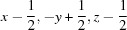
; (xi) 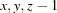; (xii) 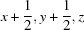.

**Table 2:** Bond-valence sum calculations for model (I) in valence units

Atom	Assumed valence state	Bond-valence sum	Deviation from assumed valence state in valence units	Bond-valence sum under consideration of mixed Fe1:Ca1 occupancy*
Ba	2	1.94	0.06	1.94
Fe1	2	1.73	0.27	2.00
Ca2	2	1.95	0.05	1.95
Al	3	2.97	0.03	2.97
F1	1	0.96	0.04	0.96
F2	1	0.99	0.01	1.03
F3	1	0.93	0.07	0.96
F4	1	1.00	0	1.00
F5	1	0.99	0.01	0.99
F6	1	0.92	0.08	0.92
F7	1	0.97	0.03	1.03

Note: (*) calculated with the weighted average Fe:Ca ratio of 0.77:0.23 at the *M*2 site.

**Table 3:** Experimental details

	Model (I)	Model (II)
Crystal data		
Chemical formula	Ba_2_Ca_1.31_Fe_0.69_Al_2_F_14_	Ba_2_CaFe_0.90_Al_2_F_14_
*M*_r_	685.68	684.98
Crystal system, space group	Monoclinic, *C*2/*c*	Monoclinic, *C*2/*c*
Temperature (K)	293	293
*a*, *b*, *c* (Å)	13.7387 (12), 5.2701 (5), 14.759 (3)	13.7387 (12), 5.2701 (5), 14.759 (3)
β (°)	92.074 (14)	92.074 (14)
*V* (Å^3^)	1067.9 (2)	1067.9 (2)
*Z*	4	4
Radiation type	Mo *K*α	Mo *K*α
μ (mm^−1^)	9.21	9.33
Crystal size (mm)	0.43 × 0.11 × 0.07	0.43 × 0.11 × 0.07

Data collection		
Diffractometer	Nonius CAD-4 four-circle diffrac­tometer	Nonius CAD-4 four-circle diffrac­tometer
Absorption correction	ψ scan (North *et al.*, 1968 ▸)	ψ scan (North *et al.*, 1968 ▸)
*T*_min_, *T*_max_	0.329, 0.901	0.329, 0.901
No. of measured, independent and observed [*I* > 2σ(*I*)] reflections	5922, 1564, 1490	5922, 1564, 1490
*R*_int_	0.055	0.055
(sin θ/λ)_max_ (Å^−1^)	0.703	0.703

Refinement		
*R*[*F* ^2^ > 2σ(*F* ^2^)], *wR*(*F* ^2^), *S*	0.032, 0.078, 1.09	0.032, 0.078, 1.10
No. of reflections	1564	1564
No. of parameters	95	96
Δρ_max_, Δρ_min_ (e Å^−3^)	2.31, −2.03	2.32, −2.03

Computer programs: *CAD-4 Software* (Enraf–Nonius, 1989 ▸), *HELENA* implemented in *PLATON* (Spek, 2009 ▸), *SHELXS97* and *SHELXL97* (Sheldrick, 2008 ▸), *ATOMS for Windows* (Dowty, 2006 ▸) and *publCIF* (Westrip, 2010 ▸).

## supplementary crystallographic information

### (modelI) Dibarium calcium iron(II) dialuminium tetradecafluoride Crystal data

**Table:**

Ba_2_CaFeAl_2_F_14_	*F*(000) = 1233
*M**_r_* = 685.68	*D*_x_ = 4.265 Mg m^−^^3^
Monoclinic, *C*2/*c*	Mo *K*α radiation, λ = 0.71073 Å
Hall symbol: -C 2yc	Cell parameters from 25 reflections
*a* = 13.7387 (12) Å	θ = 16.0–28.7°
*b* = 5.2701 (5) Å	µ = 9.21 mm^−^^1^
*c* = 14.759 (3) Å	*T* = 293 K
β = 92.074 (14)°	Lath, colourless
*V* = 1067.9 (2) Å^3^	0.43 × 0.11 × 0.07 mm
*Z* = 4	

### (modelI) Dibarium calcium iron(II) dialuminium tetradecafluoride Data collection

**Table:**

Nonius CAD-4 four-circle diffractometer	1490 reflections with *I* > 2σ(*I*)
Radiation source: fine-focus sealed tube	*R*_int_ = 0.055
Graphite monochromator	θ_max_ = 30.0°, θ_min_ = 2.8°
ω/2θ scans	*h* = −19→19
Absorption correction: ψ scan (North *et al.*, 1968)	*k* = −7→7
*T*_min_ = 0.329, *T*_max_ = 0.901	*l* = −20→20
5922 measured reflections	3 standard reflections every 240 min
1564 independent reflections	intensity decay: none

### (modelI) Dibarium calcium iron(II) dialuminium tetradecafluoride Refinement

**Table:**

Refinement on *F*^2^	Primary atom site location: isomorphous structure methods
Least-squares matrix: full	*w* = 1/[σ^2^(*F*_o_^2^) + (0.0527*P*)^2^ + 2.0371*P*] where *P* = (*F*_o_^2^ + 2*F*_c_^2^)/3
*R*[*F*^2^ > 2σ(*F*^2^)] = 0.032	(Δ/σ)_max_ < 0.001
*wR*(*F*^2^) = 0.078	Δρ_max_ = 2.31 e Å^−^^3^
*S* = 1.09	Δρ_min_ = −2.03 e Å^−^^3^
1564 reflections	Extinction correction: *SHELXL97* (Sheldrick, 2008), Fc^*^=kFc[1+0.001xFc^2^λ^3^/sin(2θ)]^-1/4^
95 parameters	Extinction coefficient: 0.0022 (2)
0 restraints	

### (modelI) Dibarium calcium iron(II) dialuminium tetradecafluoride Special details

**Table:**

Geometry. All e.s.d.'s (except the e.s.d. in the dihedral angle between two l.s. planes) are estimated using the full covariance matrix. The cell e.s.d.'s are taken into account individually in the estimation of e.s.d.'s in distances, angles and torsion angles; correlations between e.s.d.'s in cell parameters are only used when they are defined by crystal symmetry. An approximate (isotropic) treatment of cell e.s.d.'s is used for estimating e.s.d.'s involving l.s. planes.
Refinement. Refinement of *F*^2^ against ALL reflections. The weighted *R*-factor *wR* and goodness of fit *S* are based on *F*^2^, conventional *R*-factors *R* are based on *F*, with *F* set to zero for negative *F*^2^. The threshold expression of *F*^2^ > σ(*F*^2^) is used only for calculating *R*-factors(gt) *etc*. and is not relevant to the choice of reflections for refinement. *R*-factors based on *F*^2^ are statistically about twice as large as those based on *F*, and *R*- factors based on ALL data will be even larger.

### (modelI) Dibarium calcium iron(II) dialuminium tetradecafluoride Fractional atomic coordinates and isotropic or equivalent isotropic displacement parameters (Å^2^)

**Table:**

	*x*	*y*	*z*	*U*_iso_*/*U*_eq_	Occ. (<1)
Ba	0.308721 (14)	0.46574 (4)	0.379963 (13)	0.01408 (12)	
Fe1	0.0000	0.5000	0.0000	0.0122 (2)	0.690 (15)
Ca1	0.0000	0.5000	0.0000	0.0122 (2)	0.310 (15)
Ca2	0.0000	0.44616 (15)	0.2500	0.01239 (19)	
Al	0.38007 (8)	0.50390 (18)	0.12342 (7)	0.0111 (2)	
F1	0.25596 (18)	0.4521 (4)	0.0896 (2)	0.0240 (5)	
F2	0.08574 (15)	0.1721 (4)	0.97491 (13)	0.0184 (4)	
F3	0.37359 (16)	0.2100 (4)	0.55132 (14)	0.0197 (4)	
F4	0.00268 (16)	0.0837 (4)	0.15434 (15)	0.0190 (4)	
F5	0.34180 (14)	0.2933 (4)	0.71743 (13)	0.0157 (4)	
F6	0.12186 (16)	0.2677 (4)	0.80391 (15)	0.0207 (4)	
F7	0.43370 (17)	0.0715 (4)	0.37866 (16)	0.0208 (4)	

### (modelI) Dibarium calcium iron(II) dialuminium tetradecafluoride Atomic displacement parameters (Å^2^)

**Table:**

	*U*^11^	*U*^22^	*U*^33^	*U*^12^	*U*^13^	*U*^23^
Ba	0.01226 (15)	0.01401 (16)	0.01593 (15)	−0.00069 (5)	−0.00023 (9)	0.00074 (5)
Fe1	0.0106 (4)	0.0124 (3)	0.0136 (4)	0.0004 (2)	−0.0005 (3)	−0.0029 (2)
Ca1	0.0106 (4)	0.0124 (3)	0.0136 (4)	0.0004 (2)	−0.0005 (3)	−0.0029 (2)
Ca2	0.0131 (4)	0.0124 (4)	0.0118 (4)	0.000	0.0019 (3)	0.000
Al	0.0114 (5)	0.0096 (3)	0.0123 (5)	−0.0007 (3)	−0.0004 (4)	0.0005 (3)
F1	0.0130 (11)	0.0219 (11)	0.0366 (14)	−0.0017 (8)	−0.0069 (10)	−0.0007 (9)
F2	0.0218 (10)	0.0183 (9)	0.0148 (8)	0.0033 (8)	−0.0016 (7)	−0.0026 (7)
F3	0.0269 (11)	0.0140 (8)	0.0184 (9)	−0.0003 (8)	0.0027 (8)	−0.0032 (7)
F4	0.0100 (9)	0.0247 (9)	0.0224 (10)	−0.0014 (8)	0.0000 (7)	−0.0087 (8)
F5	0.0155 (9)	0.0173 (8)	0.0145 (8)	−0.0037 (7)	0.0027 (6)	0.0004 (7)
F6	0.0220 (10)	0.0161 (8)	0.0243 (10)	0.0037 (8)	0.0035 (8)	0.0076 (8)
F7	0.0183 (11)	0.0239 (10)	0.0206 (10)	0.0009 (9)	0.0052 (8)	−0.0067 (8)

### (modelI) Dibarium calcium iron(II) dialuminium tetradecafluoride Geometric parameters (Å, º)

**Table:**

Ba—F7	2.696 (2)	Ca2—F4	2.376 (2)
Ba—F4^i^	2.730 (2)	Ca2—F4^ix^	2.376 (2)
Ba—F1^i^	2.755 (2)	Ca2—F5^x^	2.544 (2)
Ba—F2^ii^	2.765 (2)	Ca2—F5^iv^	2.544 (2)
Ba—F5^iii^	2.766 (2)	Fe1—F7^x^	2.015 (2)
Ba—F5^iv^	2.827 (2)	Fe1—F7^i^	2.015 (2)
Ba—F3^iv^	2.889 (2)	Fe1—F2^xi^	2.131 (2)
Ba—F1^v^	2.889 (2)	Fe1—F2^viii^	2.131 (2)
Ba—F3	2.974 (2)	Fe1—F3^x^	2.216 (2)
Ba—F6^iii^	3.101 (2)	Fe1—F3^i^	2.216 (2)
Ba—F6^iv^	3.158 (2)	Al—F4^xii^	1.780 (2)
Ba—F1^vi^	3.233 (3)	Al—F1	1.780 (3)
Ca2—F7^vii^	2.235 (2)	Al—F6^iv^	1.790 (2)
Ca2—F7^i^	2.235 (2)	Al—F2^iv^	1.799 (2)
Ca2—F6^iii^	2.369 (2)	Al—F5^iii^	1.843 (2)
Ca2—F6^viii^	2.369 (2)	Al—F3^iii^	1.846 (2)
			
F7—Ba—F4^i^	64.22 (7)	F6^iii^—Ca2—F6^viii^	100.95 (11)
F7—Ba—F1^i^	158.04 (7)	F7^vii^—Ca2—F4	139.26 (9)
F4^i^—Ba—F1^i^	97.60 (7)	F7^i^—Ca2—F4	73.58 (8)
F7—Ba—F2^ii^	89.56 (7)	F6^iii^—Ca2—F4	133.25 (7)
F4^i^—Ba—F2^ii^	65.13 (7)	F6^viii^—Ca2—F4	109.76 (8)
F1^i^—Ba—F2^ii^	70.80 (7)	F7^vii^—Ca2—F4^ix^	73.58 (8)
F7—Ba—F5^iii^	102.89 (6)	F7^i^—Ca2—F4^ix^	139.26 (9)
F4^i^—Ba—F5^iii^	63.11 (6)	F6^iii^—Ca2—F4^ix^	109.76 (8)
F1^i^—Ba—F5^iii^	77.18 (7)	F6^viii^—Ca2—F4^ix^	133.25 (7)
F2^ii^—Ba—F5^iii^	113.14 (6)	F4—Ca2—F4^ix^	73.00 (12)
F7—Ba—F5^iv^	94.51 (6)	F7^vii^—Ca2—F5^x^	86.30 (7)
F4^i^—Ba—F5^iv^	134.23 (6)	F7^i^—Ca2—F5^x^	110.96 (8)
F1^i^—Ba—F5^iv^	107.32 (7)	F6^iii^—Ca2—F5^x^	165.39 (7)
F2^ii^—Ba—F5^iv^	159.58 (6)	F6^viii^—Ca2—F5^x^	70.35 (7)
F5^iii^—Ba—F5^iv^	85.46 (4)	F4—Ca2—F5^x^	61.35 (7)
F7—Ba—F3^iv^	108.42 (7)	F4^ix^—Ca2—F5^x^	71.46 (7)
F4^i^—Ba—F3^iv^	168.26 (6)	F7^vii^—Ca2—F5^iv^	110.96 (8)
F1^i^—Ba—F3^iv^	87.26 (7)	F7^i^—Ca2—F5^iv^	86.30 (7)
F2^ii^—Ba—F3^iv^	106.93 (6)	F6^iii^—Ca2—F5^iv^	70.35 (7)
F5^iii^—Ba—F3^iv^	128.56 (6)	F6^viii^—Ca2—F5^iv^	165.39 (7)
F5^iv^—Ba—F3^iv^	52.84 (6)	F4—Ca2—F5^iv^	71.46 (7)
F7—Ba—F1^v^	58.57 (7)	F4^ix^—Ca2—F5^iv^	61.35 (7)
F4^i^—Ba—F1^v^	122.79 (7)	F5^x^—Ca2—F5^iv^	120.53 (9)
F1^i^—Ba—F1^v^	138.03 (11)	Fe1^ix^—Ca2—Ca1^ix^	0.0
F2^ii^—Ba—F1^v^	113.81 (7)	F4^xii^—Al—F1	175.02 (12)
F5^iii^—Ba—F1^v^	128.75 (7)	F4^xii^—Al—F6^iv^	93.99 (12)
F5^iv^—Ba—F1^v^	53.35 (7)	F1—Al—F6^iv^	90.60 (11)
F3^iv^—Ba—F1^v^	50.92 (7)	F4^xii^—Al—F2^iv^	93.23 (11)
F7—Ba—F3	59.03 (6)	F1—Al—F2^iv^	88.30 (12)
F4^i^—Ba—F3	90.21 (6)	F6^iv^—Al—F2^iv^	94.65 (11)
F1^i^—Ba—F3	111.82 (7)	F4^xii^—Al—F5^iii^	87.88 (10)
F2^ii^—Ba—F3	52.19 (6)	F1—Al—F5^iii^	90.20 (12)
F5^iii^—Ba—F3	153.12 (6)	F6^iv^—Al—F5^iii^	90.11 (11)
F5^iv^—Ba—F3	113.88 (6)	F2^iv^—Al—F5^iii^	175.02 (11)
F3^iv^—Ba—F3	78.06 (7)	F4^xii^—Al—F3^iii^	88.87 (11)
F1^v^—Ba—F3	61.81 (7)	F1—Al—F3^iii^	86.45 (11)
F7—Ba—F6^iii^	149.96 (6)	F6^iv^—Al—F3^iii^	176.00 (12)
F4^i^—Ba—F6^iii^	127.50 (6)	F2^iv^—Al—F3^iii^	87.98 (11)
F1^i^—Ba—F6^iii^	50.94 (6)	F5^iii^—Al—F3^iii^	87.19 (10)
F2^ii^—Ba—F6^iii^	120.41 (6)	Al—F1—Ba^v^	114.10 (11)
F5^iii^—Ba—F6^iii^	68.78 (5)	Al—F1—Ba^i^	96.30 (9)
F5^iv^—Ba—F6^iii^	56.89 (6)	Ba^v^—F1—Ba^i^	138.03 (11)
F3^iv^—Ba—F6^iii^	63.59 (6)	Al—F1—Ba^iii^	90.11 (11)
F1^v^—Ba—F6^iii^	103.02 (6)	Ba^v^—F1—Ba^iii^	111.43 (8)
F3—Ba—F6^iii^	137.04 (6)	Ba^i^—F1—Ba^iii^	95.98 (7)
F7—Ba—F6^iv^	58.76 (6)	Al^iv^—F2—Ca1^xiii^	136.08 (11)
F4^i^—Ba—F6^iv^	67.12 (6)	Al^iv^—F2—Fe1^xiii^	136.08 (11)
F1^i^—Ba—F6^iv^	127.71 (7)	Ca1^xiii^—F2—Fe1^xiii^	0.0
F2^ii^—Ba—F6^iv^	130.73 (6)	Al^iv^—F2—Ba^xiv^	106.09 (9)
F5^iii^—Ba—F6^iv^	50.92 (6)	Ca1^xiii^—F2—Ba^xiv^	117.51 (8)
F5^iv^—Ba—F6^iv^	67.24 (5)	Fe1^xiii^—F2—Ba^xiv^	117.51 (8)
F3^iv^—Ba—F6^iv^	118.00 (6)	Al^vi^—F3—Ca1^v^	125.67 (12)
F1^v^—Ba—F6^iv^	82.68 (7)	Al^vi^—F3—Fe1^v^	125.67 (12)
F3—Ba—F6^iv^	117.62 (5)	Ca1^v^—F3—Fe1^v^	0.0
F6^iii^—Ba—F6^iv^	97.89 (5)	Al^vi^—F3—Ba^iv^	94.81 (9)
F7—Ba—F1^vi^	105.96 (6)	Ca1^v^—F3—Ba^iv^	131.32 (9)
F4^i^—Ba—F1^vi^	113.22 (7)	Fe1^v^—F3—Ba^iv^	131.32 (9)
F1^i^—Ba—F1^vi^	68.57 (8)	Al^vi^—F3—Ba	97.28 (9)
F2^ii^—Ba—F1^vi^	48.35 (6)	Ca1^v^—F3—Ba	98.63 (7)
F5^iii^—Ba—F1^vi^	144.90 (6)	Fe1^v^—F3—Ba	98.63 (7)
F5^iv^—Ba—F1^vi^	111.48 (6)	Ba^iv^—F3—Ba	101.94 (7)
F3^iv^—Ba—F1^vi^	58.64 (6)	Al^xv^—F4—Ca2	107.90 (10)
F1^v^—Ba—F1^vi^	84.02 (7)	Al^xv^—F4—Ba^v^	142.62 (11)
F3—Ba—F1^vi^	46.95 (6)	Ca2—F4—Ba^v^	109.17 (8)
F6^iii^—Ba—F1^vi^	94.27 (6)	Al^vi^—F5—Ca2^iv^	99.49 (9)
F6^iv^—Ba—F1^vi^	163.69 (5)	Al^vi^—F5—Ba^vi^	116.54 (9)
F7^x^—Fe1—F2^xi^	85.92 (8)	Ca2^iv^—F5—Ba^vi^	103.29 (7)
F7^i^—Fe1—F2^xi^	94.08 (8)	Al^vi^—F5—Ba^iv^	96.93 (8)
F7^x^—Fe1—F2^viii^	94.08 (8)	Ca2^iv^—F5—Ba^iv^	117.45 (8)
F7^i^—Fe1—F2^viii^	85.92 (8)	Ba^vi^—F5—Ba^iv^	121.50 (7)
F7^x^—Fe1—F3^x^	82.86 (8)	Al^iv^—F6—Ca2^viii^	133.22 (12)
F7^i^—Fe1—F3^x^	97.14 (8)	Al^iv^—F6—Ba^vi^	100.10 (9)
F2^xi^—Fe1—F3^x^	95.71 (8)	Ca2^viii^—F6—Ba^vi^	113.52 (8)
F2^viii^—Fe1—F3^x^	84.29 (8)	Al^iv^—F6—Ba^iv^	102.40 (9)
F7^x^—Fe1—F3^i^	97.14 (8)	Ca2^viii^—F6—Ba^iv^	101.02 (7)
F7^i^—Fe1—F3^i^	82.86 (8)	Ba^vi^—F6—Ba^iv^	102.46 (6)
F2^xi^—Fe1—F3^i^	84.29 (8)	Ca1^v^—F7—Fe1^v^	0.0
F2^viii^—Fe1—F3^i^	95.71 (8)	Ca1^v^—F7—Ca2^xvi^	120.98 (11)
F7^vii^—Ca2—F7^i^	145.63 (12)	Fe1^v^—F7—Ca2^xvi^	120.98 (11)
F7^vii^—Ca2—F6^iii^	80.29 (8)	Ca1^v^—F7—Ba	113.91 (9)
F7^i^—Ca2—F6^iii^	78.02 (8)	Fe1^v^—F7—Ba	113.91 (9)
F7^vii^—Ca2—F6^viii^	78.02 (8)	Ca2^xvi^—F7—Ba	120.86 (10)
F7^i^—Ca2—F6^viii^	80.29 (8)		

Symmetry codes: (i) −*x*+1/2, *y*+1/2, −*z*+1/2; (ii) −*x*+1/2, *y*+1/2, −*z*+3/2; (iii) *x*, −*y*+1, *z*−1/2; (iv) −*x*+1/2, −*y*+1/2, −*z*+1; (v) −*x*+1/2, *y*−1/2, −*z*+1/2; (vi) *x*, −*y*+1, *z*+1/2; (vii) *x*−1/2, *y*+1/2, *z*; (viii) −*x*, −*y*+1, −*z*+1; (ix) −*x*, *y*, −*z*+1/2; (x) *x*−1/2, −*y*+1/2, *z*−1/2; (xi) *x*, *y*, *z*−1; (xii) *x*+1/2, *y*+1/2, *z*; (xiii) *x*, *y*, *z*+1; (xiv) −*x*+1/2, *y*−1/2, −*z*+3/2; (xv) *x*−1/2, *y*−1/2, *z*; (xvi) *x*+1/2, *y*−1/2, *z*.

### (modelII) Crystal data

**Table:**

Al_2_Ba_2_CaF_14_Fe_0.90_	*F*(000) = 1240
*M**_r_* = 684.98	*D*_x_ = 4.260 Mg m^−^^3^
Monoclinic, *C*2/*c*	Mo *K*α radiation, λ = 0.71073 Å
Hall symbol: -C 2yc	Cell parameters from 25 reflections
*a* = 13.7387 (12) Å	θ = 16.0–28.7°
*b* = 5.2701 (5) Å	µ = 9.33 mm^−^^1^
*c* = 14.759 (3) Å	*T* = 293 K
β = 92.074 (14)°	Lath, colourless
*V* = 1067.9 (2) Å^3^	0.43 × 0.11 × 0.07 mm
*Z* = 4	

### (modelII) Data collection

**Table:**

Nonius CAD-4 four-circle diffractometer	1490 reflections with *I* > 2σ(*I*)
Radiation source: fine-focus sealed tube	*R*_int_ = 0.055
Graphite monochromator	θ_max_ = 30.0°, θ_min_ = 2.8°
ω/2θ scans	*h* = −19→19
Absorption correction: ψ scan (North *et al.*, 1968)	*k* = −7→7
*T*_min_ = 0.329, *T*_max_ = 0.901	*l* = −20→20
5922 measured reflections	3 standard reflections every 240 min
1564 independent reflections	intensity decay: none

### (modelII) Refinement

**Table:**

Refinement on *F*^2^	Primary atom site location: isomorphous structure methods
Least-squares matrix: full	*w* = 1/[σ^2^(*F*_o_^2^) + (0.0532*P*)^2^ + 1.6437*P*] where *P* = (*F*_o_^2^ + 2*F*_c_^2^)/3
*R*[*F*^2^ > 2σ(*F*^2^)] = 0.032	(Δ/σ)_max_ = 0.001
*wR*(*F*^2^) = 0.078	Δρ_max_ = 2.32 e Å^−^^3^
*S* = 1.10	Δρ_min_ = −2.03 e Å^−^^3^
1564 reflections	Extinction correction: *SHELXL*, Fc^*^=kFc[1+0.001xFc^2^λ^3^/sin(2θ)]^-1/4^
96 parameters	Extinction coefficient: 0.0022 (2)
0 restraints	

### (modelII) Special details

**Table:**

Geometry. All e.s.d.'s (except the e.s.d. in the dihedral angle between two l.s. planes) are estimated using the full covariance matrix. The cell e.s.d.'s are taken into account individually in the estimation of e.s.d.'s in distances, angles and torsion angles; correlations between e.s.d.'s in cell parameters are only used when they are defined by crystal symmetry. An approximate (isotropic) treatment of cell e.s.d.'s is used for estimating e.s.d.'s involving l.s. planes.
Refinement. Refinement of *F*^2^ against ALL reflections. The weighted *R*-factor *wR* and goodness of fit *S* are based on *F*^2^, conventional *R*-factors *R* are based on *F*, with *F* set to zero for negative *F*^2^. The threshold expression of *F*^2^ > σ(*F*^2^) is used only for calculating *R*-factors(gt) *etc*. and is not relevant to the choice of reflections for refinement. *R*-factors based on *F*^2^ are statistically about twice as large as those based on *F*, and *R*- factors based on ALL data will be even larger.

### (modelII) Fractional atomic coordinates and isotropic or equivalent isotropic displacement parameters (Å^2^)

**Table:**

	*x*	*y*	*z*	*U*_iso_*/*U*_eq_	Occ. (<1)
Ba	0.308719 (14)	0.46574 (4)	0.379962 (12)	0.01408 (12)	
Ca	0.0000	0.44617 (15)	0.2500	0.01239 (19)	
Al	0.38008 (8)	0.50390 (18)	0.12340 (7)	0.0111 (2)	
Fe	0.0000	0.5000	0.0000	0.0114 (3)	0.901 (5)
F1	0.25596 (18)	0.4521 (4)	0.0896 (2)	0.0240 (5)	
F2	0.08572 (15)	0.1721 (4)	0.97492 (13)	0.0184 (4)	
F3	0.37360 (16)	0.2100 (4)	0.55132 (14)	0.0197 (4)	
F4	0.00267 (15)	0.0837 (4)	0.15434 (15)	0.0190 (4)	
F5	0.34178 (14)	0.2932 (4)	0.71742 (13)	0.0157 (4)	
F6	0.12185 (16)	0.2677 (4)	0.80392 (14)	0.0207 (4)	
F7	0.43372 (16)	0.0715 (4)	0.37867 (16)	0.0208 (4)	

### (modelII) Atomic displacement parameters (Å^2^)

**Table:**

	*U*^11^	*U*^22^	*U*^33^	*U*^12^	*U*^13^	*U*^23^
Ba	0.01226 (16)	0.01399 (16)	0.01592 (15)	−0.00069 (5)	−0.00023 (9)	0.00074 (5)
Ca	0.0131 (4)	0.0124 (4)	0.0118 (4)	0.000	0.0019 (3)	0.000
Al	0.0114 (5)	0.0096 (3)	0.0123 (5)	−0.0007 (3)	−0.0003 (4)	0.0005 (3)
Fe	0.0098 (4)	0.0115 (3)	0.0128 (4)	0.0004 (2)	−0.0005 (3)	−0.0030 (2)
F1	0.0129 (11)	0.0219 (11)	0.0366 (14)	−0.0017 (8)	−0.0068 (10)	−0.0007 (9)
F2	0.0219 (10)	0.0184 (9)	0.0148 (8)	0.0032 (8)	−0.0016 (7)	−0.0026 (7)
F3	0.0270 (11)	0.0140 (8)	0.0184 (9)	−0.0004 (8)	0.0027 (8)	−0.0031 (7)
F4	0.0100 (9)	0.0247 (9)	0.0224 (10)	−0.0013 (8)	0.0000 (7)	−0.0086 (8)
F5	0.0156 (9)	0.0172 (8)	0.0145 (8)	−0.0038 (7)	0.0027 (6)	0.0004 (7)
F6	0.0221 (10)	0.0160 (8)	0.0243 (10)	0.0037 (8)	0.0036 (8)	0.0076 (8)
F7	0.0182 (11)	0.0238 (9)	0.0207 (10)	0.0008 (8)	0.0053 (8)	−0.0067 (8)

### (modelII) Geometric parameters (Å, º)

**Table:**

Ba—F7	2.696 (2)	Ca—F4	2.376 (2)
Ba—F4^i^	2.730 (2)	Ca—F4^ix^	2.376 (2)
Ba—F1^i^	2.755 (2)	Ca—F5^x^	2.544 (2)
Ba—F2^ii^	2.766 (2)	Ca—F5^iv^	2.544 (2)
Ba—F5^iii^	2.767 (2)	Fe—F7^i^	2.015 (2)
Ba—F5^iv^	2.826 (2)	Fe—F7^x^	2.015 (2)
Ba—F3^iv^	2.889 (2)	Fe—F2^viii^	2.131 (2)
Ba—F1^v^	2.889 (2)	Fe—F2^xi^	2.131 (2)
Ba—F3	2.974 (2)	Fe—F3^x^	2.216 (2)
Ba—F6^iii^	3.101 (2)	Fe—F3^i^	2.216 (2)
Ba—F6^iv^	3.159 (2)	Al—F4^xii^	1.779 (2)
Ba—F1^vi^	3.232 (3)	Al—F1	1.780 (3)
Ca—F7^vii^	2.235 (2)	Al—F6^iv^	1.790 (2)
Ca—F7^i^	2.235 (2)	Al—F2^iv^	1.799 (2)
Ca—F6^iii^	2.369 (2)	Al—F5^iii^	1.843 (2)
Ca—F6^viii^	2.369 (2)	Al—F3^iii^	1.846 (2)
			
F7—Ba—F4^i^	64.22 (7)	F7^i^—Ca—F4^ix^	139.26 (9)
F7—Ba—F1^i^	158.04 (7)	F6^iii^—Ca—F4^ix^	109.75 (8)
F4^i^—Ba—F1^i^	97.60 (7)	F6^viii^—Ca—F4^ix^	133.25 (7)
F7—Ba—F2^ii^	89.56 (7)	F4—Ca—F4^ix^	73.01 (12)
F4^i^—Ba—F2^ii^	65.12 (7)	F7^vii^—Ca—F5^x^	86.30 (7)
F1^i^—Ba—F2^ii^	70.80 (7)	F7^i^—Ca—F5^x^	110.95 (8)
F7—Ba—F5^iii^	102.89 (6)	F6^iii^—Ca—F5^x^	165.38 (7)
F4^i^—Ba—F5^iii^	63.12 (6)	F6^viii^—Ca—F5^x^	70.34 (7)
F1^i^—Ba—F5^iii^	77.18 (7)	F4—Ca—F5^x^	61.35 (7)
F2^ii^—Ba—F5^iii^	113.14 (6)	F4^ix^—Ca—F5^x^	71.47 (7)
F7—Ba—F5^iv^	94.51 (6)	F7^vii^—Ca—F5^iv^	110.95 (7)
F4^i^—Ba—F5^iv^	134.23 (6)	F7^i^—Ca—F5^iv^	86.30 (7)
F1^i^—Ba—F5^iv^	107.33 (6)	F6^iii^—Ca—F5^iv^	70.34 (7)
F2^ii^—Ba—F5^iv^	159.58 (6)	F6^viii^—Ca—F5^iv^	165.38 (7)
F5^iii^—Ba—F5^iv^	85.46 (4)	F4—Ca—F5^iv^	71.47 (7)
F7—Ba—F3^iv^	108.42 (6)	F4^ix^—Ca—F5^iv^	61.35 (7)
F4^i^—Ba—F3^iv^	168.26 (6)	F5^x^—Ca—F5^iv^	120.54 (9)
F1^i^—Ba—F3^iv^	87.26 (7)	F4^xii^—Al—F1	175.02 (12)
F2^ii^—Ba—F3^iv^	106.93 (6)	F4^xii^—Al—F6^iv^	94.00 (11)
F5^iii^—Ba—F3^iv^	128.56 (6)	F1—Al—F6^iv^	90.60 (11)
F5^iv^—Ba—F3^iv^	52.85 (6)	F4^xii^—Al—F2^iv^	93.23 (11)
F7—Ba—F1^v^	58.57 (7)	F1—Al—F2^iv^	88.31 (12)
F4^i^—Ba—F1^v^	122.79 (7)	F6^iv^—Al—F2^iv^	94.65 (11)
F1^i^—Ba—F1^v^	138.03 (11)	F4^xii^—Al—F5^iii^	87.88 (10)
F2^ii^—Ba—F1^v^	113.81 (7)	F1—Al—F5^iii^	90.18 (12)
F5^iii^—Ba—F1^v^	128.76 (7)	F6^iv^—Al—F5^iii^	90.11 (11)
F5^iv^—Ba—F1^v^	53.36 (6)	F2^iv^—Al—F5^iii^	175.02 (10)
F3^iv^—Ba—F1^v^	50.92 (7)	F4^xii^—Al—F3^iii^	88.87 (11)
F7—Ba—F3	59.02 (6)	F1—Al—F3^iii^	86.45 (11)
F4^i^—Ba—F3	90.21 (6)	F6^iv^—Al—F3^iii^	175.99 (12)
F1^i^—Ba—F3	111.81 (7)	F2^iv^—Al—F3^iii^	87.98 (10)
F2^ii^—Ba—F3	52.19 (6)	F5^iii^—Al—F3^iii^	87.19 (10)
F5^iii^—Ba—F3	153.12 (6)	F7^i^—Fe—F2^viii^	85.92 (8)
F5^iv^—Ba—F3	113.88 (6)	F7^x^—Fe—F2^viii^	94.08 (8)
F3^iv^—Ba—F3	78.06 (7)	F7^i^—Fe—F2^xi^	94.08 (8)
F1^v^—Ba—F3	61.81 (6)	F7^x^—Fe—F2^xi^	85.92 (8)
F7—Ba—F6^iii^	149.97 (6)	F7^i^—Fe—F3^x^	97.13 (8)
F4^i^—Ba—F6^iii^	127.50 (6)	F7^x^—Fe—F3^x^	82.87 (8)
F1^i^—Ba—F6^iii^	50.94 (6)	F2^viii^—Fe—F3^x^	84.30 (8)
F2^ii^—Ba—F6^iii^	120.42 (6)	F2^xi^—Fe—F3^x^	95.70 (8)
F5^iii^—Ba—F6^iii^	68.77 (5)	F7^i^—Fe—F3^i^	82.87 (8)
F5^iv^—Ba—F6^iii^	56.89 (6)	F7^x^—Fe—F3^i^	97.13 (8)
F3^iv^—Ba—F6^iii^	63.59 (6)	F2^viii^—Fe—F3^i^	95.70 (8)
F1^v^—Ba—F6^iii^	103.02 (6)	F2^xi^—Fe—F3^i^	84.30 (8)
F3—Ba—F6^iii^	137.04 (6)	Al—F1—Ba^v^	114.10 (11)
F7—Ba—F6^iv^	58.77 (6)	Al—F1—Ba^i^	96.30 (9)
F4^i^—Ba—F6^iv^	67.12 (6)	Ba^v^—F1—Ba^i^	138.03 (10)
F1^i^—Ba—F6^iv^	127.71 (7)	Al—F1—Ba^iii^	90.11 (11)
F2^ii^—Ba—F6^iv^	130.72 (6)	Ba^v^—F1—Ba^iii^	111.43 (8)
F5^iii^—Ba—F6^iv^	50.93 (5)	Ba^i^—F1—Ba^iii^	95.98 (7)
F5^iv^—Ba—F6^iv^	67.24 (5)	Al^iv^—F2—Fe^xiii^	136.08 (11)
F3^iv^—Ba—F6^iv^	118.00 (6)	Al^iv^—F2—Ba^xiv^	106.08 (9)
F1^v^—Ba—F6^iv^	82.69 (7)	Fe^xiii^—F2—Ba^xiv^	117.52 (8)
F3—Ba—F6^iv^	117.62 (5)	Al^vi^—F3—Fe^v^	125.66 (11)
F6^iii^—Ba—F6^iv^	97.89 (5)	Al^vi^—F3—Ba^iv^	94.81 (9)
F7—Ba—F1^vi^	105.96 (6)	Fe^v^—F3—Ba^iv^	131.32 (9)
F4^i^—Ba—F1^vi^	113.22 (7)	Al^vi^—F3—Ba	97.27 (9)
F1^i^—Ba—F1^vi^	68.57 (8)	Fe^v^—F3—Ba	98.64 (7)
F2^ii^—Ba—F1^vi^	48.36 (6)	Ba^iv^—F3—Ba	101.94 (7)
F5^iii^—Ba—F1^vi^	144.89 (5)	Al^xv^—F4—Ca	107.92 (10)
F5^iv^—Ba—F1^vi^	111.48 (6)	Al^xv^—F4—Ba^v^	142.62 (11)
F3^iv^—Ba—F1^vi^	58.64 (6)	Ca—F4—Ba^v^	109.17 (8)
F1^v^—Ba—F1^vi^	84.02 (7)	Al^vi^—F5—Ca^iv^	99.48 (9)
F3—Ba—F1^vi^	46.95 (6)	Al^vi^—F5—Ba^vi^	116.53 (9)
F6^iii^—Ba—F1^vi^	94.27 (6)	Ca^iv^—F5—Ba^vi^	103.28 (7)
F6^iv^—Ba—F1^vi^	163.69 (5)	Al^vi^—F5—Ba^iv^	96.94 (8)
F7^vii^—Ca—F7^i^	145.64 (12)	Ca^iv^—F5—Ba^iv^	117.45 (7)
F7^vii^—Ca—F6^iii^	80.29 (8)	Ba^vi^—F5—Ba^iv^	121.51 (7)
F7^i^—Ca—F6^iii^	78.04 (8)	Al^iv^—F6—Ca^viii^	133.22 (12)
F7^vii^—Ca—F6^viii^	78.04 (8)	Al^iv^—F6—Ba^vi^	100.10 (9)
F7^i^—Ca—F6^viii^	80.29 (8)	Ca^viii^—F6—Ba^vi^	113.52 (8)
F6^iii^—Ca—F6^viii^	100.95 (11)	Al^iv^—F6—Ba^iv^	102.40 (9)
F7^vii^—Ca—F4	139.26 (9)	Ca^viii^—F6—Ba^iv^	101.01 (7)
F7^i^—Ca—F4	73.57 (8)	Ba^vi^—F6—Ba^iv^	102.45 (6)
F6^iii^—Ca—F4	133.25 (7)	Fe^v^—F7—Ca^xvi^	120.99 (11)
F6^viii^—Ca—F4	109.75 (8)	Fe^v^—F7—Ba	113.91 (9)
F7^vii^—Ca—F4^ix^	73.57 (8)	Ca^xvi^—F7—Ba	120.85 (10)

Symmetry codes: (i) −*x*+1/2, *y*+1/2, −*z*+1/2; (ii) −*x*+1/2, *y*+1/2, −*z*+3/2; (iii) *x*, −*y*+1, *z*−1/2; (iv) −*x*+1/2, −*y*+1/2, −*z*+1; (v) −*x*+1/2, *y*−1/2, −*z*+1/2; (vi) *x*, −*y*+1, *z*+1/2; (vii) *x*−1/2, *y*+1/2, *z*; (viii) −*x*, −*y*+1, −*z*+1; (ix) −*x*, *y*, −*z*+1/2; (x) *x*−1/2, −*y*+1/2, *z*−1/2; (xi) *x*, *y*, *z*−1; (xii) *x*+1/2, *y*+1/2, *z*; (xiii) *x*, *y*, *z*+1; (xiv) −*x*+1/2, *y*−1/2, −*z*+3/2; (xv) *x*−1/2, *y*−1/2, *z*; (xvi) *x*+1/2, *y*−1/2, *z*.
